# Endogenous antibodies contribute to macrophage-mediated demyelination in a mouse model for CMT1B

**DOI:** 10.1186/s12974-015-0267-y

**Published:** 2015-03-12

**Authors:** Dennis Klein, Janos Groh, Andreas Weishaupt, Rudolf Martini

**Affiliations:** Department of Neurology, Developmental Neurobiology, University Hospital Würzburg, Josef-Schneider-Str 11, D-97080 Würzburg, Germany

**Keywords:** Charcot-Marie-Tooth, Demyelination, Antibodies, Macrophages, Adaptive immune system, B-lymphocytes, Macrophages, Fc-receptor, Complement

## Abstract

**Background:**

We could previously identify components of both the innate and the adaptive immune system as disease modifiers in the pathogenesis of models for Charcot-Marie-Tooth (CMT) neuropathies type 1B and 1X. As part of the adaptive immune system, here we investigated the role of antibodies in a model for CMT1B.

**Methods:**

Antibodies were localized and characterized in peripheral nerves of the CMT1B model by immunohistochemistry and Western blot analysis. Experimental ablation of antibodies was performed by cross breeding the CMT1B models with mutants deficient in B-lymphocytes (JHD−/− mutants). Ameliorated demyelination by antibody deficiency was reverted by intravenous injection of mouse IgG fractions. Histopathological analysis was performed by immunocytochemistry and light and quantitative electron microscopy.

**Results:**

We demonstrate that in peripheral nerves of a mouse model for CMT1B, endogenous antibodies strongly decorate endoneurial tubes of peripheral nerves. These antibodies comprise IgG and IgM subtypes and are preferentially, but not exclusively, associated with nerve fiber aspects nearby the nodes of Ranvier. In the absence of antibodies, the early demyelinating phenotype is substantially ameliorated. Reverting the neuropathy by reconstitution with murine IgG fractions identified accumulating antibodies as potentially pathogenic at this early stage of disease.

**Conclusions:**

Our study demonstrates that in a mouse model for CMT1B, endogenous antibodies contribute to early macrophage-mediated demyelination and disease progression. Thus, both the innate and adaptive immune system are mutually interconnected in a genetic model for demyelination. Since in Wallerian degeneration antibodies have also been shown to be involved in myelin phagocytosis, our study supports our view that inherited demyelination and Wallerian degeneration share common mechanisms, which are detrimental when activated under nonlesion conditions.

## Background

Charcot-Marie-Tooth (CMT) type 1 disorders comprise a genetically heterogeneous group of inherited peripheral neuropathies that are characterized by length-dependent axonal degeneration, muscle atrophy, and sensory dysfunction, substantially reducing quality of life [1,2]. Although in the last years several culprit genes could be identified, presently no causative treatment is available [3,4].

Our group could identify low-grade inflammation as a substantial disease modifier in the pathogenesis of distinct CMT1 mouse models [5]. While in models for CMT1A, macrophages were identified as the only inflammation-related disease modulators [6,7], disease outcome in models for CMT1B and CMT1X is influenced by components of both the innate [8-12] and the adaptive immune system [13-15].

Based on the finding that antibodies, a humoral component of the adaptive immune system, are involved in myelin phagocytosis after peripheral nerve lesion [16] and due to putative molecular similarities between Wallerian degeneration and pathogenesis of CMT [5], we investigated the role of antibodies in a demyelinating model for CMT1B.

We demonstrate here that endoneurial tubes of peripheral nerves of P0het myelin mutant mice are decorated with endogenous antibodies. Furthermore, by crossbreeding P0het mice with mouse mutants specifically lacking B-lymphocytes and antibodies (JHD−/−), we show a significant amelioration of demyelination and a reduction of macrophages in peripheral nerves of young P0het JHD−/− mice. Passive systemic transfer of antibodies (IgGs) into P0het JHD−/− mice restored antibody decoration and reverted reduced macrophage elevation and impaired nerve histology, suggesting a role of endogenous antibodies in macrophage-mediated demyelination. Our study therefore identifies endogenous antibodies as a link in the interaction between the innate and adaptive immune system during early pathogenesis in a model for CMT1B.

## Methods

### Animals

Mice heterozygously deficient for P0 (P0het; [17]) were crossbred to animals specifically lacking B-lymphocytes (JHD−/−; [18]) according to previously published protocols [13] and investigated at the age of 1 and 6 months with the corresponding littermates of both gender. As additional controls, RAG1-deficient mice, lacking both B- and T-lymphocytes, were analyzed [19]. All mice were on a C57BL/6 N background, and genotypes were identified after purification of genomic DNA from ear biopsies using DNeasy Blood & Tissue Kit (Qiagen, Venlo, The Netherlands) according to the manufacturer’s guidelines. Genotypes of P0 mutants were determined by conventional PCR using oligonucleotides 5′-TCAGTTCCTTGTCCCCCGCTCTC-3′, 5′-GGCTGCAGGGTCGCTCGGTGTTC-3′, and 5′-ACTTGTCTCTTCTGGGTAATCAA-3′ leading to 334 or 500 bp products for the P0 null mutation or wildtype allele; genotypes of JHD mutants with oligonucleotides 5′-GAGGAGACGGTGACCGTGGTCCCTGC-3′, 5′-GGACCAGGGGGCTCAGGTCACTC-AGG-3′, 5′-GCCGCATTGCATCAGCCATGAT-GGA-3′, and 5′-CCTTGCGCAGCTGTGC-TCGACGTTG-3′ leading to 180 or 195 bp products for the JHD null mutation or wildtype allele; genotypes of RAG1 mutants with oligunucleotides 5′-GAGGTTCCGCTACGACTCTG-3′, 5′-CCGGACAAGTTTTTCAT-CGT-3′, and 5′-TGGATGTGGAATTGTTGCGAG-3′ leading to 530 or 474 bp products for the RAG1 null mutation or wildtype allele, respectively. Animals were kept in the animal facility of the Department of Neurology, University clinic of Würzburg, in a 12 h/12 h day (<300 lux)/night rhythm under barrier conditions using individually ventilated cages. All animal experiments were approved by the local authority, the Government of Lower Franconia, Germany.

### Sciatic nerve crush injury

Wildtype mice were deeply anesthetized by an intraperitoneal injection of a mixture of ketamine and xylazine (10 μl per g body weight). The right sciatic nerve was exposed and crushed at the region of the sciatic notch by using a non-serrated clamp using a constant pressure for 15 s. This resulted in a completely translucent appearance of the crushed area of the nerve. After 3 days, the mice were sacrificed and lesioned nerves were removed for analysis of complement deposition (see below).

### Tissue processing

For preparation of fresh frozen spleens and femoral nerves, animals were killed by asphyxiation with CO_2_ (according to guidelines by the State Office of Health and Social Affairs Berlin), blood was transcardially rinsed with phosphate-buffered saline (PBS) containing heparin, followed by harvesting and embedding nerves in O.C.T. medium (Sakura, Alphen aan den Rijn, The Netherlands) and frozen in liquid nitrogen-cooled methyl butane.

For preparation of fixed femoral nerves, blood of sacrificed mice (see above) was transcardially rinsed with PBS/heparin followed by perfusion with 4% paraformaldehyde (PFA) in PBS for 10 min. Dissected nerves were post-fixed in the same solution for 2 h, rinsed in 30% sucrose/PBS overnight at 4°C, and embedded in O.C.T. medium. Peripheral nerves were cut into 10 μm-thick cross sections on a cryostat (Leica, Solms, Germany) and stored at −20°C.

Single teased-fiber preparations were processed as described elsewhere [20]. Briefly, blood was transcardially rinsed with PBS/heparin followed by perfusion with 2% PFA in PBS for 10 min. Femoral quadriceps nerves were dissected, and single fibers were separated by forceps on glass slides.

For electron microscopy, femoral quadriceps nerves were processed as described previously [11]. In short, mice were transcardially perfused with 4% PFA and 2% glutaraldehyde in 0.1 M cacodylate buffer. Dissected nerves were post-fixed in the same solution overnight at 4°C. After osmification and dehydration, samples were embedded in Spurr’s medium. Ultrathin sections (80 nm) were mounted to copper grids and counterstained with lead citrate.

### Immunohistochemistry

For identification of endogenous antibodies on nerve cross sections or teased fibers, PFA-fixed samples were blocked with 10% bovine serum albumin (BSA) and 1% normal goat serum (NGS) in 0.1 M PBS, followed by incubation with Cy3-conjugated goat-anti-mouse IgG-Fc antibodies (1:300, 715-166-150, Dianova, Hamburg, Germany) in 1% BSA and 1% NGS in 0.1 M PBS for 1 h at room temperature. Alternatively, samples were incubated overnight with non-coupled rabbit-anti-mouse IgG-Fc (1:1,000, 31194, Thermo Scientific, Waltham, MA, USA) at 4°C, followed by incubation with the corresponding Cy3-conjugated secondary antibodies for 1 h (1:300, 111-165-144, Dianova). To determine whether endogenous nerve antibodies are bound to extra- or intracellular domains, unfixed and non-permeabilized native sciatic nerves were loosely teased and incubated free floating in a 96-well plate. IgG-Fc staining was performed similarly as described above. To control for internal antibody deposition, nerve fibers were permeabilized by repeated cycles of freezing in liquid nitrogen and thawing. Incubation with rabbit-anti-mouse β-III-Tubulin (1:500, ab18207, Abcam, Cambridge, UK) and detection with the corresponding Cy3-conjugated secondary antibodies for 1 h (1:300, 111-165-144, Dianova) were additionally performed and served as positive controls for successful permeabilization and internal antibody binding.

Complement deposition was identified on teased fiber preparations. Briefly, samples were fixed in acetone (10 min, −20°C), blocked with 5% NGS with 0.3% TritonX-100 in 0.1 M PBS, and incubated with FITC-conjugated goat-mouse C3 (1:100, 0855500, MP Biomedicals, Santa Ana, CA, USA) in 1% NGS with 0.3% TritonX-100 in 0.1 M PBS for 1 h at room temperature. As positive controls, teased fibers from distal parts of lesioned sciatic nerves (3 days after crush) were identically treated as uninjured nerves of mutant mice. Diaphragms, incubated *ex vivo* with anti-ganglioside monoclonal antibodies and normal human serum [21], were investigated for complement deposition as lesioned nerves, whereby postsynaptic terminals were identified with Alexa Fluor 555-conjugated α-Bungarotoxin (1:300, B35451, Molecular Probes, Life Technologies, Carlsbad, CA, USA).

Quantification of endoneurial macrophages was performed on cross sections according to previously published protocols [8,11]. Briefly, fresh-frozen samples were fixed in acetone (10 min, −20°C) and blocked with 5% BSA in 0.1 M PBS, followed by an avidin-biotin blocking step (SP-2001, Vector Laboratories, Burlingame, CA, USA); biotinylated rat-anti-F4/80 (1:300, MCA497B, Serotec, Kidlington, UK) primary antibodies in 1% BSA in 0.1 M PBS were applied for 1 h at room temperature and detected by Cy3-conjugated Streptavidin (1:100, CED-CLCSA1010, Biozol, Eching, Germany). Nuclei were labeled with DAPI (Sigma-Aldrich, St. Louis, MO, USA). Whole nerve cross sections were analyzed, and the mean number of macrophages per section in seven to ten consecutive sections per animal was calculated.

Determination of B-lymphocytes was performed on cross sections of the spleen. In short, fresh-frozen samples were fixed in acetone (10 min, −20°C), blocked with 5% BSA in 0.1 M PBS and incubated with rat-anti-B220/CD45R (1:100, 550286, BD Pharmingen, San Jose, CA, USA) primary antibodies in 1% BSA in 0.1 M PBS overnight at 4°C, and detected by Cy3-conjugated secondary antibodies (1:300, 112-165-167, Dianova).

Digital fluorescence microscopic images were acquired using an Axiophot 2 microscope (Zeiss, Oberkochen, Germany) equipped with a CCD camera (Visitron Systems, Puchheim, Germany) and afterwards processed with Photoshop CS3 (Adobe, San Jose, CA, USA). IgG fluorescence intensity in the endoneurium of femoral quadriceps nerve cross sections was measured with ImageJ (NIH, Bethesda, Maryland). Briefly, images were converted to 32-bit grayscale images and the mean gray value was calculated to determine immunoreactive signals, taking the mean gray value of P0wt nerves as a reference value. Data is shown as the mean of *n* = 2 per genotype of three repeated stainings and measurements. At least three sections per animal and experiment were analyzed.

### Western blot analysis

Determination of protein concentrations and Western blot analysis were performed as previously described [11]. Briefly, animals were killed by asphyxiation with CO_2_, blood was rinsed with PBS/heparin, and femoral quadriceps nerves were quickly dissected and snap frozen in liquid nitrogen. After sonification (Sonoplus HD60, Bandelin Electronic, Berlin, Germany) in 100 μL RIPA lysis buffer per 10 mg tissue, protein concentration was determined by a Lowry assay (Sigma-Aldrich). Proteins were resolved by sodium dodecyl sulfate-polyacrylamide.

After gel electrophoresis (SDS-PAGE with 10% Acrylamide), proteins were transferred to a nitrocellulose membrane and visualized with Ponceau S (Roth, Newport Beach, CA, USA). Membranes were blocked with 5% skimmed milk in PBST and incubated with rabbit-anti-ERK1/2 (1:10,000, sc-94, Santa Cruz Biotechnology, Dallas, TX, USA) antibody solution overnight at 4°C. Corresponding horseradish peroxidase (HRP)-conjugated secondary antibodies (1:3,000, NA9340V, GE Healthcare, Little Chalfont, UK) were probed for 1 h at room temperature, and detection of the immune reaction was determined by ECL reagent and ECL hyperfilm (GE Healthcare Bio-Sciences AB). Endogenous antibodies were detected by directly labeled HRP-anti mouse IgG-Fc antibodies (1:5,000, 115-035-008, Dianova) overnight at 4°C or for 2 h at room temperature. Densitometric analyses were performed with Image J (NIH) and depicted as the ratio of IgG to ERK1/2. Results from two independent experiments are shown.

### Morphological analysis

Multiple image alignments were acquired, and abnormally myelinated fibers, consisting of thinly and demyelinated axons, onion bulbs, and foamy macrophages were quantified in relation to the total number of axons in cross sections of the femoral quadriceps nerve. The g-ratio was determined by dividing the diameter of the axon by the diameter of the same axon including its myelin sheath. At least 125 fibers per animal were randomly selected and analyzed. Analysis was performed using a ProScan Slow Scan CCD camera mounted to a Leo 906E electron microscope (Zeiss) with corresponding iTEM software (Olympus Soft Imaging Solutions GmbH, Münster, Germany).

### Reconstitution experiments

150 μg of mouse IgGs (PMP01, Serotec) or mouse-anti-keyhole limpet hemocyanin (KLH)-antibody (orb11067, Biorbyt, Cambridge, UK) or 50 μg mouse IgG Fc fragments (31205, Pierce, Rockford, IL, USA) were injected into the tail vein (i.v.) once or at four consecutive weeks followed by analysis 1 week later. After dissection and tissue processing, specific binding of antibodies in peripheral nerves was controlled by immunohistochemistry and Western blot analysis.

### Statistical analysis

All experiments were performed in a blinded manner, with the investigators unaware of the genotypes of the analyzed mice. Data sets were controlled for normal distribution by Shapiro-Wilk test. Comparison of more than two groups with normally distributed data (parametric) was tested by one-way ANOVA, followed by Tukey *post hoc* test or Bonferroni-Holm correction. In the case of non-normally distributed data sets, the nonparametric Kruskal-Wallis test with Bonferroni-Holm correction was applied. Significance levels (*, # *P* < 0.05; **, ## *P* < 0.01; ***, ### *P* < 0.001) are indicated together with the applied statistical tests within the figure legends. Quantifications are shown as mean + SD. Statistical analyses were performed using PASW Statistics 18 (SPSS, IBM, Armonk, NY, USA) software, and diagrams were generated with Microsoft Excel 2007.

## Results

### Antibodies accumulate in peripheral nerves of P0het myelin mutant mice

**Figure 1:**
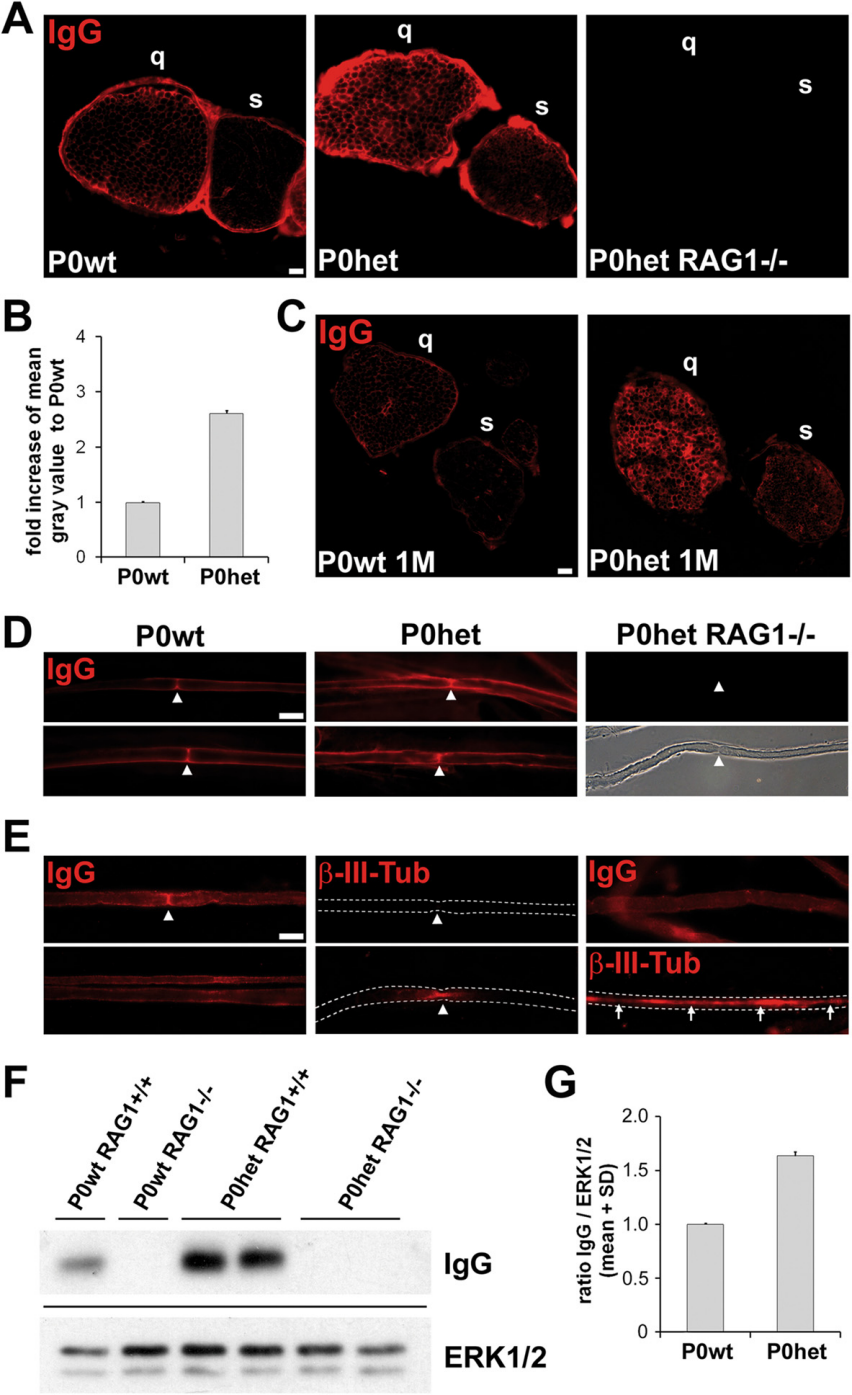
**Antibodies accumulate in peripheral nerves of myelin mutant mice. (A)** Immunohistochemical localization of endogenous antibodies (IgG) in the femoral quadriceps (q) and saphenous nerve (s) from 6-month-old wildtype mice (P0wt) and myelin mutants (P0het). Note the prominent labeling of the endoneurium in nerve cross sections in femoral quadriceps nerves of mutants in contrast to wildtype mice. RAG1-deficient mice (deficient for T- and B-lymphocytes) are, expectedly, devoid of antibody deposition. Scale bar, 20 μm. **(B)** IgG fluorescence intensity in the endoneurium of femoral quadriceps nerve cross sections is shown as the mean gray value, taking P0wt nerves as a reference. *n* = 2 animals per genotype were analyzed within three repeated stainings and measurements. **(C)** Immunohistochemical localization of endogenous antibodies (IgG) in the femoral quadriceps (q) and saphenous nerve (s) from 1-month-old wildtype mice (P0wt) and myelin mutants (P0het). Note similar endoneurial immunoreactivity in comparison to 6-month-old mutants (see **A**), but the lower intensity of the perineurium. Scale bar, 20 μm. **(D)** Immunocytochemical staining against IgG on single fiber preparations. Note preferential deposition of antibodies in the nodal regions and the generally stronger signal along myelinated fibers in P0het mice compared to wildtype mice. Nerve-specific antibodies are not detectable in RAG1-deficient mice. The corresponding phase contrast micrograph displays the teased fiber with a node of Ranvier (arrowheads). Scale bar, 20 μm. **(E)** Immunocytochemical staining against IgG or β-III-Tubulin on unfixed and non-permeabilized native sciatic nerve fibers of P0het mice (left and middle column). Note IgG immunoreactivity along the outer margins of the myelinated nerve fibers and at nodes of Ranvier (arrowhead, left column) whereas β-III-Tubulin-immunoreactivity is confined to some axonal profiles at nodes of Ranvier (arrowheads, middle column), likely due to the vulnerability of this structure upon teasing. In the permeabilized sciatic nerve samples of P0het mice, β-III-Tubulin immunoreactivity is now visible along the entire axon (arrows, lower right column) and no longer confined to nodes (see middle columns), whereas IgG immunoreactivity is still detectable along myelinated fibers (upper right column). White dashed lines indicate outer margins of myelinated nerve fibers. Scale bar, 20 μm. **(F)** Western blot analysis of wildtype (P0wt) and P0het femoral quadriceps nerve lysates showing stronger IgG signal in mutant mice. RAG1-deficient mice show no antibody signal. ERK1/2 serves as a loading control. **(G)** Densitometric analysis of IgG immunoreactivity in Western blots from P0het mutant mice and P0wt littermates, taking ERK1/2 signal as a reference (loading control). Results of *n* = 2 to 3 from two independent experiments are depicted.

In order to detect antibody binding, we performed immunohistochemical stainings for IgGs on femoral nerve cross sections from 6-month-old P0het mutant mice and P0wt littermates. We found a strong antibody deposition in the perineurium of femoral quadriceps nerves of both P0wt and P0het mutants (Figure 1A). However, in the endoneurium of mutant nerves, a higher IgG immunoreactivity was observed compared to wildtype controls which could be confirmed by IgG signal intensity measurements (Figure 1A,B). Of note, in the non-affected sensory branch of the femoral nerve (saphenous nerve), this prominent antibody deposition could not be detected in P0het mutant mice (Figure 1A). We also performed IgG immunocytochemistry on single teased-fiber preparations, in order to detect potentially preferred antibody depositions along myelinated nerve fibers. Indeed, we could confirm a stronger deposition along myelinated fibers in P0het mice compared to wildtype controls, with strongest IgG immunoreactivity at nodes of Ranvier (Figure 1D). To determine whether endogenous nerve antibodies are bound to extra- or intracellular domains, nerve fibers of unfixed and non-permeabilized sciatic nerves of P0het mice were loosely teased and stained free floating for IgG and β-III-Tubulin. Again, IgG immunoreactivity could be detected along myelinated nerve fibers indicating external deposition of endogenous antibodies, whereas β-III-Tubulin immunoreactivity was confined to some axonal profiles at nodes of Ranvier (Figure 1E), likely reflecting the vulnerability of this structure by mechanical teasing. As controls for the accessibility of intracellular domains by permeabilization, nerve fibers were repeatedly frozen in liquid nitrogen followed by incubation with antibodies to IgGs or β-III-Tubulin. As expected, only after permeabilization, β-III-Tubulin staining showed an extended immunoreactivity along the entire axon including internodes, while IgG immunoreactivity was still visible along the outer margins of the myelinated fibers (Figure 1E). Thus, endogenous antibodies bind to extracellular domains of peripheral nerve fibers.

In order to substantiate our immunocytochemical findings, we performed Western blot analysis of peripheral nerve extracts using antibodies to mouse IgG and IgM subtypes. We found increased IgG (Figure 1F,G) and IgM signals (not shown) in P0het mice in comparison to extracts from P0wt mice, corroborating the immunocytochemical observations. Specificity of the respective staining was further controlled by performing immunocytochemistry and Western blot analysis in RAG1-deficient mice, lacking T- and B-lymphocytes (and, therefore, antibodies). As expected, this resulted in a complete lack of decorating antibodies in peripheral nerves (Figure 1). We also investigated antibody deposition in 1-month-old P0het mice and P0wt littermates, an early time point in disease before features typical for demyelination are morphologically visible. Indeed, by immunohistochemistry (Figure 1C) and Western blot analysis (not shown), we detected already at this early age a similarly elevated IgG immunoreactivity in the mutant nerves as at 6 months of age. However, perineurial staining was substantially weaker.

Our data suggest that specific endogenous antibodies bind to extracellular domains of endoneurial tubes in peripheral nerves of myelin mutant mice, a potential early prerequisite for modulating pathogenesis in an animal model for CMT1B.

### Lack of evidence for complement deposition

**Figure 2:**
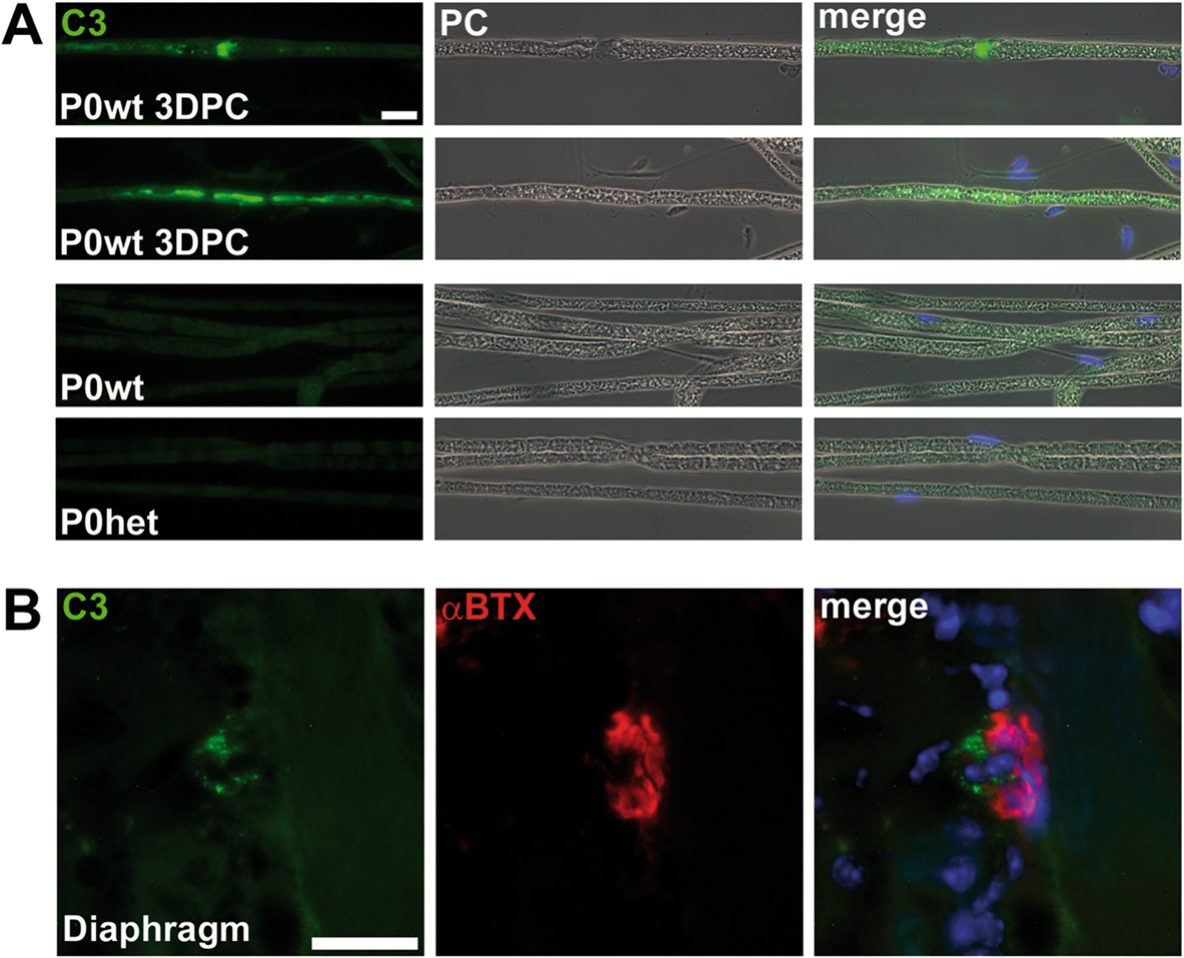
**Lack of evidence for complement deposition in peripheral nerve fibers of P0het mice. (A)** Immunocytochemical staining against C3 on single teased-fiber preparations. Complement deposition is detectable in teased fibers from crushed wildtype sciatic nerves (3 days post crush) but not in nonlesioned sciatic nerves from 6-month-old P0wt and P0het mice. Corresponding phase contrast (PC) micrographs are also shown. **(B)** Immunohistochemical staining against C3 (green) and α-Bungarotoxin (αBTX, red) on diaphragm cross section. Complement deposition is detectable at presynaptic terminals. Blue profiles in merged micrographs are DAPI-labeled nuclei. Scale bars, 5 μm.

Deposition of antibodies is often associated with the classical or alternative pathways of complement activation [22,23]. We, therefore, investigated whether in our P0het mice, C3, a central component of both pathways, is deposited on mutant nerve fibers. By immunohistochemistry using a C3-specific antibody, we failed to identify complement deposition, while - as positive controls - C3 deposition was amply detectable on nerve fibers of crushed nerves [24] and on explanted diaphragms incubated with anti-ganglioside antibodies and normal human serum [21] (Figure 2).

### Reduced macrophage numbers in peripheral nerves of P0het JHD−/− mice

**Figure 3:**
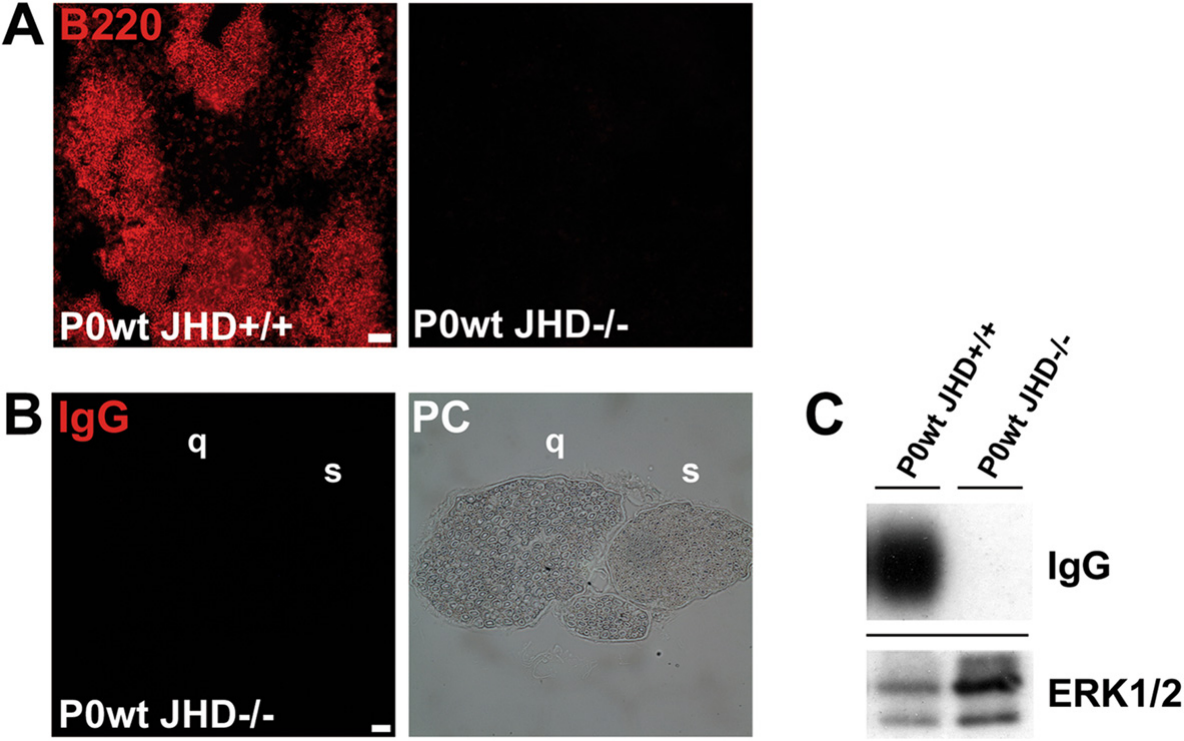
**JHD-deficient mice lack B-lymphocytes and antibodies. (A)** Immunohistochemical staining against B220-positive B-lymphocytes in wildtype controls and JHD-deficient mice. Note lack of immunoreactivity in JHD-deficient mice. Scale bar, 50 μm. **(B)** IgG deposition was not detectable in 6-month-old femoral quadriceps (q) and saphenous (s) nerves from JHD-deficient mice. The corresponding phase contrast (PC) microscope image is also shown. Scale bar, 20 μm. **(C)** Western blot analysis confirms the lack of endogenous antibodies in JHD-deficient mice. ERK1/2 serves as a loading control.

In order to identify the role of antibodies in a model for CMT1B, we crossbred P0het mice with JHD-deficient mutants that lack B-lymphocytes and are, thus, incapable to produce antibodies. As expected, JHD−/− mice were devoid of B-lymphocytes in the spleen and antibodies in peripheral nerves as revealed by immunohistochemistry and Western blot analysis (Figure 3).

**Figure 4:**
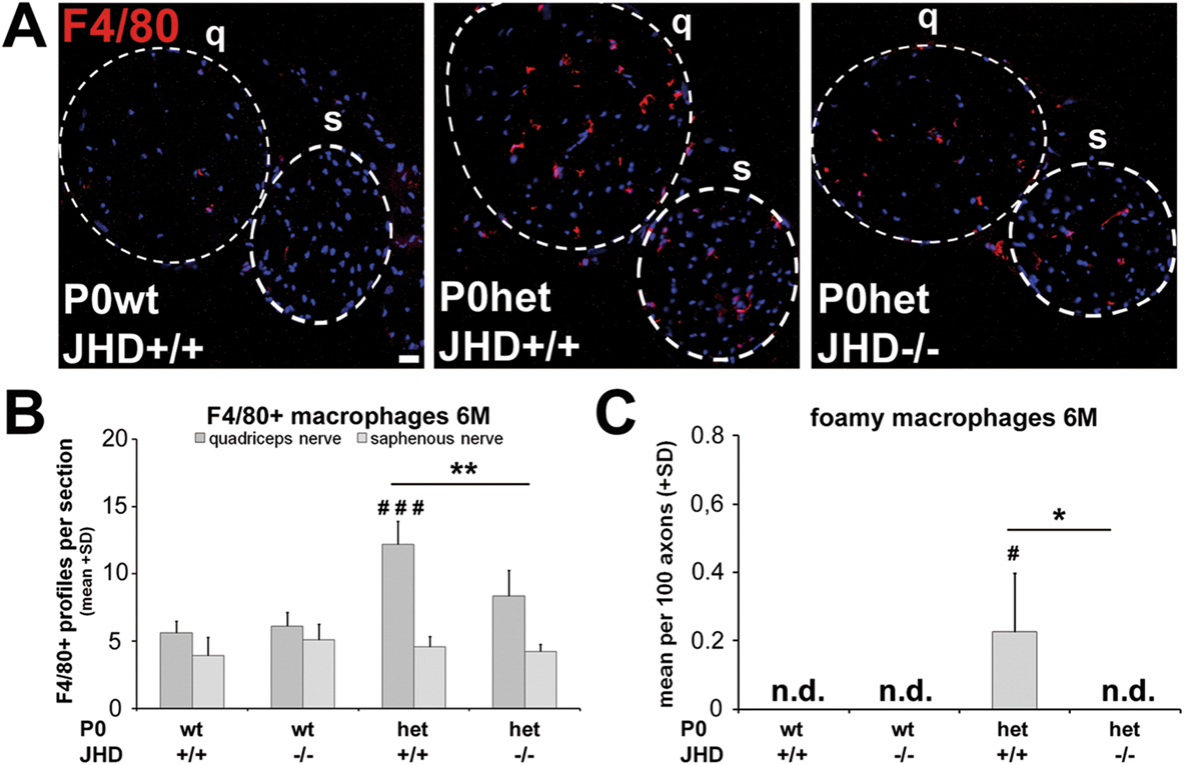
**Reduced macrophage numbers in P0het mice lacking endogenous antibodies. (A)** Immunohistochemistry against macrophages (F4/80) in cross sections of femoral nerves from 6-month-old wildtype (P0wt JHD+/+), single (P0het JHD+/+), and double mutants (P0het JHD−/−). Representative micrographs are shown. Dashed circles indicate the femoral quadriceps (q) and saphenous nerve (s). Scale bar, 20 μm. **(B)** Quantification of F4/80-positive profiles showing a significant reduction of macrophage numbers in femoral quadriceps nerve from P0het JHD−/− double mutants in comparison to single mutants. In the saphenous nerve, the number of macrophages is not increased in mutants (*n* = 4 to 5). One-way ANOVA with Tukey *post hoc* test. # (significant difference to P0wt), * (significant difference between P0het mutant groups). #, *P < 0.05; ##, **P < 0.01; ###, ***P < 0.001. **(C)** Quantification of foamy macrophages reveals an absence of phagocytosing macrophages in femoral quadriceps nerves from P0het JHD−/− mice (*n* = 4 to 5). Kruskal-Wallis test with Bonferroni-Holm correction. # (significant difference to P0wt), * (significant difference between P0het mutant groups). #, *P < 0.05. n.d. (not detected).

Next, we investigated the number of macrophages in P0wt and P0het mice either positive or homozygously deficient for JHD (Figure 4). Numbers of macrophages were significantly elevated in femoral quadriceps nerves of 6-month-old P0het mice when compared to age-matched wildtype mice, confirming previous observations [8,9]. Interestingly, P0het mice additionally deficient for JHD and lacking endogenous antibodies showed a significantly attenuated elevation of F4/80-positive macrophages (Figure 4A,B). However, in pathologically non-affected saphenous nerves, the number of macrophages was not altered in single and double mutant mice (Figure 4A,B). We additionally focused on macrophage activation by electron microscopy. We found a substantial number of macrophages only in P0het mice that were JHD positive, whereas all other genotypes, including P0het JHD−/− mice, lacked foamy macrophages (Figure 4C).

### Antibody deficiency ameliorates the demyelinating phenotype in P0het mutant mice

**Figure 5:**
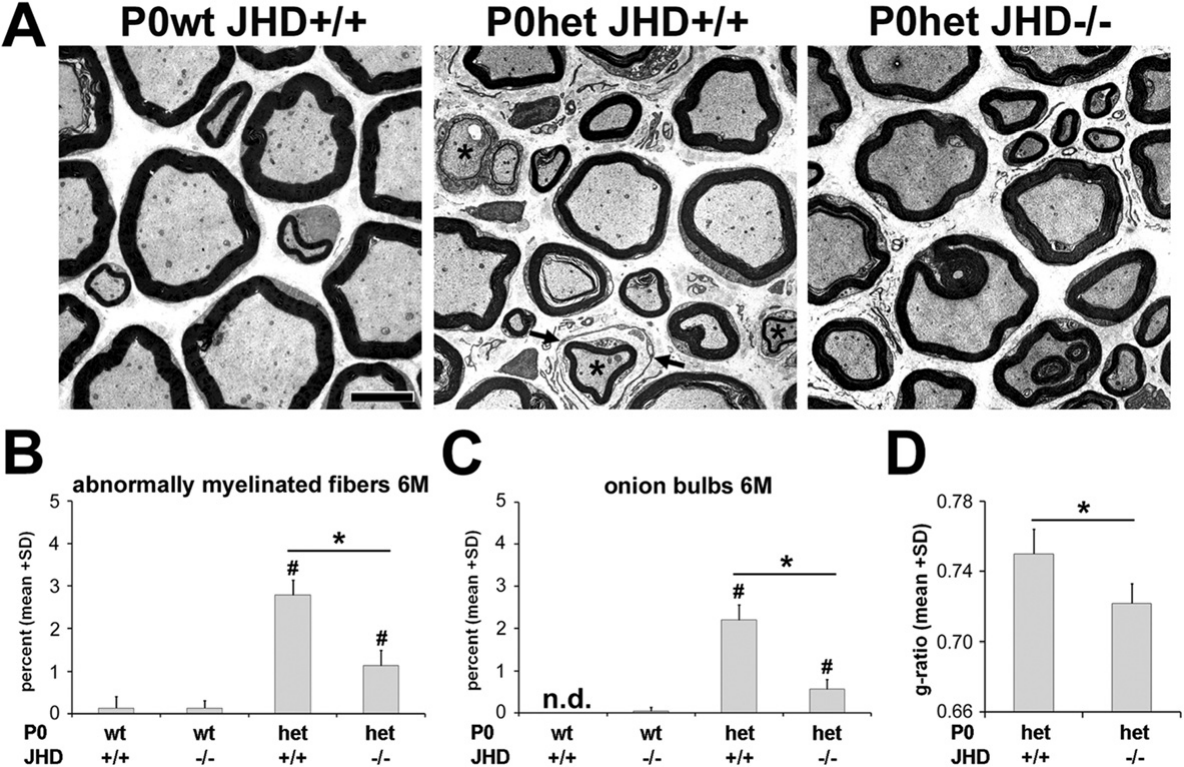
**Antibody deficiency ameliorates myelin degeneration in P0het mutants. (A)** Representative ultrathin sections of femoral quadriceps nerves from 6-month-old wildtype (P0wt JHD+/+), single (P0het JHD+/+), and double mutants (P0het JHD−/−). Asterisks indicate abnormally myelinated fibers and arrows supernumerary Schwann cells (‘onion bulbs’). Scale bar, 5 μm. **(B,** **C)** Quantification of abnormally myelinated fibers **(B)** and onion bulbs **(C)** demonstrate signs of significant amelioration of demyelination and improved myelin integrity in P0het JHD−/− double mutants in comparison to P0het JHD+/+ mice (*n* = 4 to 5). Kruskal-Wallis test with Bonferroni-Holm correction, # (significant difference to P0wt), * (significant difference between P0het mutant groups). #, *P < 0.05. n.d. (not detected). **(D)** Quantification of the g-ratio (axon diameter/fiber diameter) demonstrates a significant increase in myelin thickness in P0het JHD−/− compared to P0het JHD+/+ mice (*n* = 4, 125 to 135 axons per animal). Two-tailed Student’s *t*-test. *P < 0.05.

As a next step, we investigated whether absence of antibodies in P0het JHD−/− mice leads to an alleviation of demyelination, a typical feature of our CMT1B mouse model. Quantification by electron microscopy of profiles indicative of pathological alterations revealed a significant reduction of abnormally myelinated fibers and of supernumerary Schwann cells (‘onion bulbs’) in P0het JHD−/− mice compared to P0het JHD+/+ mice at 6 months of age (Figure 5). Moreover, quantification of the g-ratio (axon diameter/fiber diameter) showed a significant increase in myelin thickness in P0het JHD−/− compared to P0het JHD+/+ mice (Figure 5D) demonstrating an improved myelin integrity. Thus, endogenous antibodies seem to contribute to early macrophage-mediated demyelination in P0het myelin mutant mice.

### Passive transfer of antibodies reverts the ameliorated demyelinating phenotype in P0het JHD−/− mice and suggests antigen specificity

**Figure 6:**
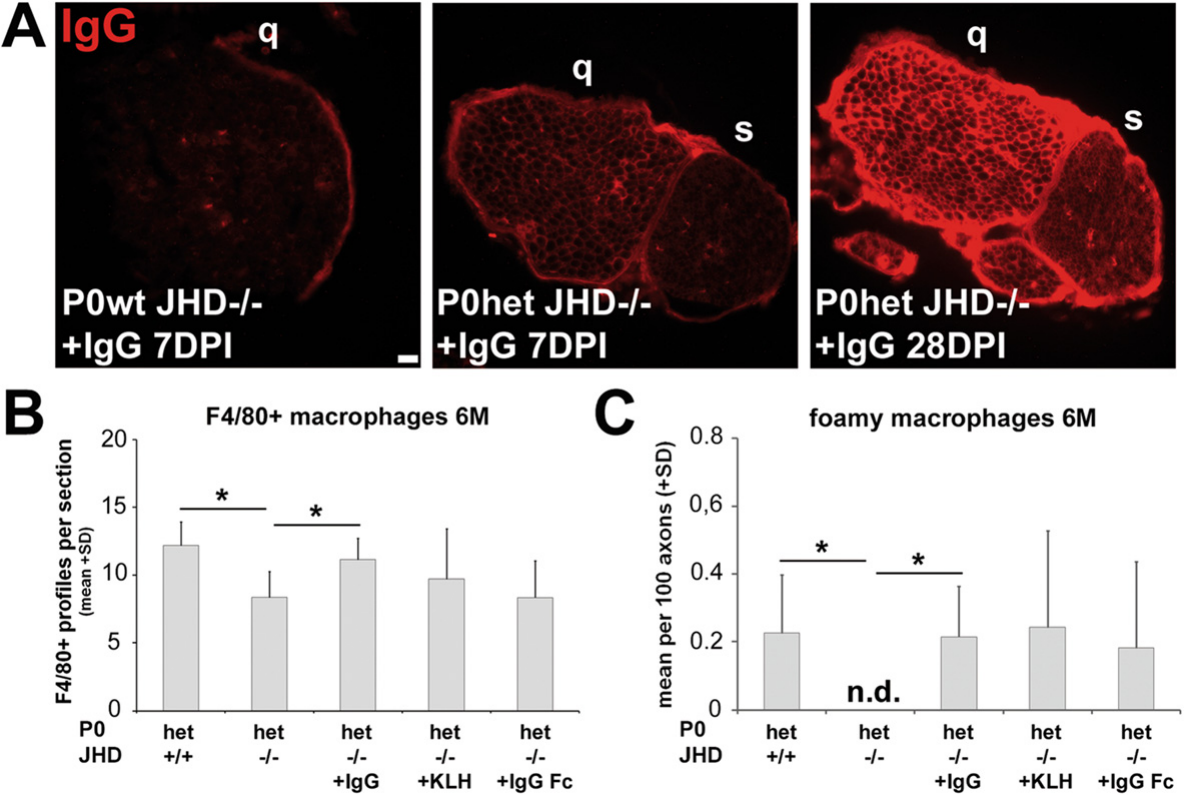
**Antibodies accumulate in peripheral nerves after passive transfer and lead to macrophage increase. (A)** Immunohistochemical localization of antibodies after systemic injection of IgG in the femoral quadriceps (q) and saphenous nerve (s) from 6-month-old P0wt JHD−/− (7 days post injection, DPI) and P0het JHD−/− mice (7DPI and 28DPI). Note stronger IgG immunoreactivity in the endoneurium from P0het JHD−/− mice that received consecutive IgG injections over 28 days (28DPI). Scale bar, 20 μm. **(B)** Quantification of F4/80-positive profiles in non-injected control and reconstituted mice showing an increase of macrophage numbers in femoral quadriceps nerve from P0het JHD−/− mutants after IgG reconstitution (*n* = 3 to 5). One-way ANOVA with Bonferroni-Holm correction. *P < 0.05. **(C)** Quantification of foamy macrophages reveals an increase of phagocytosing macrophages in femoral quadriceps nerves from P0het JHD−/− mice after IgG reconstitution. Note also the presence of phagocytosing macrophages in control-reconstituted animals (+KLH, +IgG Fc) (*n* = 3 to 5). Kruskal-Wallis test with Bonferroni-Holm correction. *P < 0.05. n.d. (not detected).

**Figure 7:**
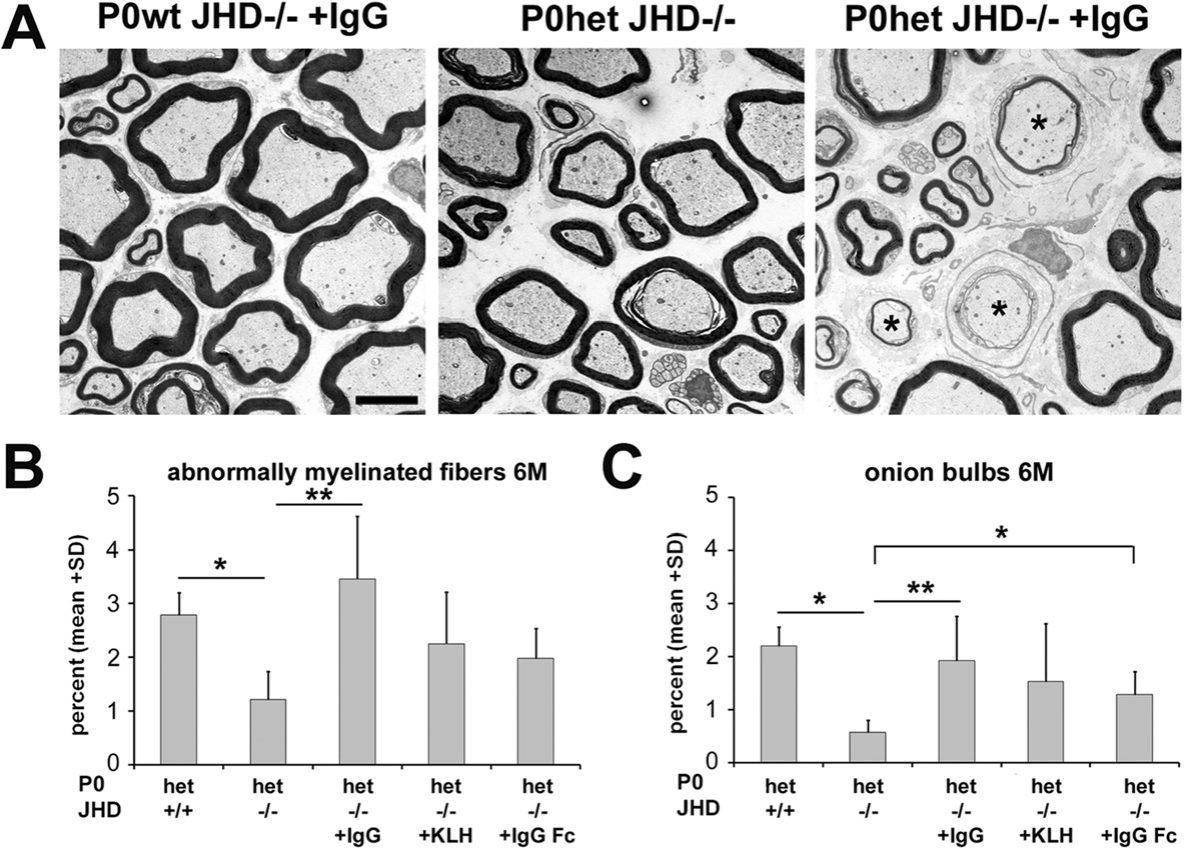
**Antibody reconstitution reverts the ameliorated demyelinating phenotype in P0het JHD−/− mice. (A)** Representative ultrathin sections of femoral quadriceps nerves from 6-month-old IgG reconstituted P0wt JHD−/−, P0het JHD+/+ controls, and IgG reconstituted P0het JHD−/− mice. Asterisks indicate abnormally myelinated fibers. Scale bar, 5 μm. **(B, C)** Quantification of abnormally myelinated fibers **(B)** and signs of demyelination (onion bulbs) **(C)** in control and reconstituted mice reveal a significantly aggravated myelin degeneration in IgG reconstituted P0het JHD−/− mutants. Note also a non-significant trend of increased pathological alterations in control-reconstituted animals (+KLH, +IgG Fc). (*n* = 3 to 5). Kruskal-Wallis test with Bonferroni-Holm correction. *P < 0.05; **P < 0.01.

In order to clarify the role of endogenous antibodies in the pathogenesis in a model for CMT1B, we performed reconstitution experiments into P0het JHD−/− mice to determine if antibodies can revert the observed beneficial effects. We performed i.v. injections of IgGs either once or on four consecutive weeks and analyzed mice at the age of 6 months. By immunohistochemistry, we found that after passive transfer into P0het JHD−/− mice, IgGs accumulated on endoneurial tubes of peripheral nerves comparable to P0het JHD+/+ mice, although to a weaker degree (Figure 6A). IgG deposition was increased in femoral quadriceps nerves after four weekly injections compared to mice that received only a single IgG injection (Figure 6A). The passive transfer led not only to increased antibody detection in nerve sections but also to elevated numbers of F4/80-positive macrophages and foamy macrophages in P0het JHD−/− mice, comparable to P0het JHD+/+ mutants (Figure 6B,C), whereas antibody reconstitution had no effect on numbers of putative resident macrophages in P0wt JHD−/− mice (not shown). To analyze the pathogenic impact of injected antibodies, we quantified nerve fiber damage by electron microscopy. P0wt JHD−/− showed no pathological alterations after IgG injections (Figure 7A). However, IgG injections reverted the ameliorated phenotype of P0het JHD−/− mice, as determined by an increased number of abnormally myelinated fibers and supernumerary Schwann cells (Figure 7B,C), suggesting that antibodies are sufficient to aggravate nerve damage along with macrophage-mediated demyelination.

Finally, we investigated whether antigen specificity is needed for the detrimental effect of antibodies. We performed passive transfer experiments into P0het JHD−/− mice using mouse antibodies that show specificity for a non-mammalian antigen, like keyhole limpet hemocyanin (KLH), or mouse-IgG Fc fragments. Expectedly, in both cases, we failed to demonstrate antibody binding to peripheral nerves by immunohistochemistry (not shown). Unexpectedly, we could observe a slight, non-significant increase of total macrophage numbers after injection of non-specific IgGs or mouse-IgG Fc fragments in individual animals (Figure 6B) together with an increase of phagocytosing macrophages (Figure 6C). Consistent with this macrophage activation, we could also detect slightly increased nerve fiber damage after reconstitution (Figure 7), suggesting that antibodies not binding to neural tissue can activate macrophages and contribute to demyelination in peripheral nerves of P0het mice.

## Discussion

In the present work, we demonstrate that in peripheral nerves of P0het mice, a model for CMT1B, endogenous antibodies strongly decorate endoneurial tubes of peripheral nerves whereas nerve fibers of wt mice are only weakly labeled. These antibodies comprise IgG and IgM subtypes and are preferentially, but not exclusively, associated with nerve fiber aspects nearby nodes of Ranvier. Furthermore, in the absence of antibodies, the early demyelinating phenotype is substantially ameliorated. Reverting the neuropathy by reconstitution with murine IgG fractions identified accumulating antibodies as potentially pathogenic at this early stage of disease.

Antibody deposition as an accelerator of myelin phagocytosis has recently been demonstrated for nerve injury, inducing Wallerian degeneration [16]. After crush injury, endogenous, preexisting antibodies were strongly accumulated along endoneurial tubes of the lesioned as opposed to intact nerves, where similarly weak labeling was detected as in our wt control mice. At least a subpopulation of these systemic antibodies has been identified to recognize the myelin component P0/MPZ. Based on the similar staining patterns obtained and the systemic presence of such antibodies in every normal mouse, it is very likely that the antibodies binding to the endoneurial tubes of our CMT1B model share the same specificities. It is, however, presently not known why preexisting antibodies do not accumulate to unlesioned wt nerves. It is likely that the intact blood-nerve barrier is one explanation [16]. Additionally, it is plausible to assume that epitopes ‘hidden’ in normal myelin are only accessible under pathological conditions. An argument in favor of the latter hypothesis is that we failed to demonstrate an interrupted blood-nerve barrier in our young CMT1B models (Martini, unpublished).

In an approach using JHD−/− mice, lack of antibodies led to lower macrophage numbers and mildly delayed myelin removal by the phagocytes in the lesion model. Additionally, likely as a consequence of prolonged persistence of myelin debris [25], axonal regeneration was delayed in the absence of antibodies in the injured nerves [16]. Thus, antibodies have a beneficial function under lesion conditions, whereas in the CMT model, antibodies foster demyelination of initially almost normal nerve fibers which are typical for young CMT1B mutants. This supports our view that in inherited demyelination, similar mechanisms are working as in Wallerian degeneration but lead to neural dysfunction when activated under nonlesion conditions [5].

How might antibody deposition and macrophage-related demyelination be interconnected?

Phagocytotic performance of macrophages involves a broad range of receptors for recognition, binding, and incorporation, such as receptors for immunoglobulins and complement or certain selectins [26]. The best known receptors for macrophages are complement receptor 3 (CR3; also known as αMβ2, Mac-1 or CD11b) and the Fcγ receptor [26-28]. Since the complement component C3 could not be found associated to mutant nerve fibers or their subcellular domains, we consider CR3 as being involved in antibody-driven pathogenesis as unlikely. By contrast, the macrophage-related Fcγ receptor, amply expressed on nerve macrophages (unpublished observation), might be an important mediator in functionally linking the innate with the adaptive immune system in our models.

Interestingly, CSF-1, a nerve fibroblast-derived cytokine that has pivotal activating impact on phagocytosing macrophages in our models [8,11,29] has been reported to be involved in Fcγ-receptor upregulation in macrophages under various pathological conditions [30]. Thus, it is plausible to assume that CSF-1 not only leads to intrinsic macrophage proliferation and activation [29] but additionally ‘prepares’ macrophages for recognition of antibody depositions.

Reconstitution experiments in P0het JHD−/− mice using murine IgG antibodies reverted the beneficial effect of antibody deficiency. Interestingly, reconstitution with antibodies specific to a non-mammalian antigen, KLH, or with Fc fragments from murine IgG generated an ‘intermediate’ effect in that abnormally myelinated axons and onion bulbs were elevated in individual animals in comparison to non-reconstituted P0het JHD−/− mice. Although the corresponding values in the reconstituted vs. non-reconstituted double mutants were not statistically significantly different from each other, these data may suggest that both antibodies bound to endoneurial tubes and antibodies not target bound might have the capacity to contribute to macrophage activation.

We have shown that, in an early stage of genetically-induced demyelination, systemic, preexisting antibodies link the adaptive and innate immune system during the macrophage-mediated demyelinating process. Of particular note is the preferential deposition of antibodies in the femoral quadriceps as opposed to the saphenous nerve, as the latter nerve is preserved from myelin degeneration and inflammation [31,32]. It is possible that binding of antibodies might contribute to MCP-1 and - indirectly - CSF-1 expression in quadriceps nerves which are important activators of macrophages in our models [5]. However, in the absence of antibodies, myelin destruction is not as prominently blocked as with MCP-1 reduction [9] or CSF-1 deficiency [8]. This might imply that antibodies at early stages of disease might have an amplifying rather than causative role of myelin degeneration in quadriceps nerves of P0het mice.

Surprisingly, at older ages, JHD deficiency has obviously a diametrically opposite effect in P0het mice as they reveal increased macrophage activation and subsequent aggravated demyelination. Likely, based on our unpublished observations, this effect is maybe due to altered cytokine profiles caused by B-lymphocyte deprivation. Indeed, B-lymphocyte deficiency triggers an alternative macrophage response in 12-month-old P0het mice as measured by qRT-PCR (Klein, Martini unpublished), similar to observations after acute nerve injury [33]. Taken together, it is presently difficult to decide whether the absence of antibodies or the altered cytokine profile due to B-lymphocyte deficiency aggravates the phenotype at older ages.

In the present study, we provide evidence for a pathogenic role of antibodies in a model for a distinct inherited demyelinating neuropathy. Interestingly, in some vigorous forms of CMT, IVIG treatment was helpful [34]. However, it is presently difficult to estimate whether the observations in these case reports can be taken as a hint of a role of endogenous antibodies in the patients, since IVIGs fulfill multiple functions at various levels of the innate and adaptive immune system [35,36]. In summary, our study extends our understanding of pathomechanisms possibly involved in inherited demyelinating neuropathies in humans. Moreover, the present passive transfer approach of specific antibodies into P0het JHD−/− mice may provide an experimental setup to test identified human pathogenic antibodies in detail with regard to their preferential subcellular target site in diseased peripheral nerves [37,38].
